# Harm Reduction Practices and Needs in a Belgian Chemsex Context: Findings from a Qualitative Study

**DOI:** 10.3390/ijerph17239081

**Published:** 2020-12-04

**Authors:** Corinne Herrijgers, Karolien Poels, Heidi Vandebosch, Tom Platteau, Jacques van Lankveld, Eric Florence

**Affiliations:** 1 Department of Clinical Sciences, Institute of Tropical Medicine, 2000 Antwerp, Belgium; TPlatteau@itg.be (T.P.); eflorence@itg.be (E.F.); 2 Department of Communication Studies, Faculty of Social Sciences, University of Antwerp, 2000 Antwerp, Belgium; karolien.poels@uantwerpen.be (K.P.); heidi.vandebosch@uantwerpen.be (H.V.); 3 Department of Psychology, Open University, 6400 Heerlen, The Netherlands; Jacques.vanLankveld@ou.nl

**Keywords:** chemsex, GBMSM, harm reduction, needs, mobile health intervention, high-risk behavior

## Abstract

Chemsex is a growing public health concern, with little evidence-based care and support available. The aim of this study is to understand current risk reduction practices, and the information and care needs of gay, bisexual, and other men who have sex with men (GBMSM) who engage in chemsex. Between January and March 2020, semi structured in-depth interviews with drug-using GBMSM (*n* = 20) were conducted. Data were analyzed thematically. The reported preparatory measures were: deliberately scheduling chemsex sessions, and discussing preferences regarding setting and attendees. During the event, a logbook is kept to monitor drugs taken by each participant. People try to take care of each other, but this is often counteracted. Respondents highlighted needs: reliable and easily-accessible information, anonymous medical and psychological healthcare, chemsex-specific care, and a value-neutral safe space to talk about chemsex experiences. Results imply two types of users: planned and impulsive users. Adherence to intended harm reduction practices are complicated by drug effects, peer pressure, and feelings of distrust among users.

## 1. Introduction

In recent years, chemsex has emerged as a public health concern among gay, bisexual, and other men who have sex with men (GBMSM) [1,2]. The term refers to the use of drugs in a sexual context [3,4], and is limited to the GBMSM community and the distinct set of cultural factors that are specific to gay sex [5]. These factors include society’s lack of acceptance, the impact of the HIV epidemic, internalized homophobia, coming-out issues, experiences of discrimination and violence, and normalization of drug use in the gay community [4]. The concept of chemsex must be understood from a syndemic perspective, as a complex phenomenon, with its specific behaviors, underlying issues, and characteristics, of a high-risk group of GBMSM [4,6,7,8]. Reasons for drug use and chemsex are manifold, but can be reinforced by feelings of loneliness, sense of shame and stigma, lack of self-confidence, and lack of meaningful connections [8]. Several definitions of chemsex have been formulated as the drugs used may vary according to specific locations and time periods [6]. Generally, based on research in the United Kingdom (UK), the following drugs are associated with chemsex: crystal methamphetamine, mephedrone, gamma-butyrolactone (GBL), and gamma-hydroxybutyric acid (GHB) [4,7]. A study on chemsex in Brussels (*n* = 362) shows that cocaine, ecstasy, and ketamine are also commonly used in this context [8]. This finding was confirmed during the interviews, as respondents also described the use of drugs such as ecstasy, cocaine, ketamine, and new psychoactive substances (NPS) to intensify the total sexual experience. Therefore, we decided to include these four drugs in our definition of chemsex [9,10]. Alcohol, cannabis, and poppers are generally not included, and were therefore also excluded from our definition [10]. Geo-spatial dating apps (e.g., Grindr, Scruff) are often used to facilitate access to sexual partners and drugs [4,11]. Chemsex can take place between two people (“chemdate”), but usually involves more participants. Chemsex sessions are primarily organized in people’s (private) homes and, to a lesser extent at gay-specific sex-on-premises venues [12]. This study is limited to the context of chemsex events in people’s homes. The respondents did not mention participating in chemsex at bars, saunas, sex clubs, or other venues. The sessions can last from a few hours to several days [4,13,14].

While the use of drugs may start from a hedonistic perspective [15] and allow GBMSM to engage in the kinds of sex they desire [16], a number of studies have associated chemsex with a variety of sex- and drug-specific health harms. Drug related health harms are wide ranging, including: drug overdose, psychosis, dehydration, hyperthermia, drug-induced violence and injuries, drug dependence, etc. [17]. Drug use is also associated with lower adherence to HIV medication (antiretroviral drugs), which may result in therapy failure and STI transmission [18]. Moreover, numerous studies have shown that polydrug use is the norm in this context, as only a few participants use only one drug [19,20,21]. Using several drugs exposes the individual to even higher risks due to the combined effects [17,22]. Understanding the specific harms associated with polydrug use is challenging, because it encompasses a wide variety of drug combinations. In general, polydrug use is associated with a higher risk of drug overdose [22], persistence of drug abuse disorders [23], and increased risk of psychological harm [24]. In this context, studies have found interactions with HIV medication and erectile dysfunction medications containing sildenafil (Viagra™), such as the potentiation of sildenafil and the increased likelihood of experiencing adverse effects (headache, flushing, and hypotension) [25]. In addition to these drug related harms, certain sexual behaviors increase the risk of HIV/STI transmission, such as condomless anal intercourse, having a large number of sexual partners, sharing sex toys, having prolonged sexual sessions, and higher risk sexual practices (e.g., fisting) [3,26,27,28]. These behaviors can lead to sexually transmitted infections, HIV [27,29,30] and/or blood-borne viruses (e.g., hepatitis C, syphilis, gonorrhea) [3]. Adverse mental health outcomes, such as anxiety and depression, have also been increasingly reported [31,32].

The European Internet Survey (EMIS), carried out in 2017, shows that 11% of Belgian GBMSM respondents have used drugs to make sex more intense or last longer, making it the second highest proportion of respondents (after The Netherlands) [33]. The first report that focused on the Belgian epidemiological chemsex context was published by Van Acker [8]. This explorative quantitative study (*n* = 362) was limited to the Brussels region, and was aimed at understanding the specificities of chemsex, regarding drug consumption patterns, sexual preferences, the contexts in which they occur, and understanding chemsex related health harms. This study showed that more than one in four respondents encountered psychological problems (e.g., depression, mood swings, paranoia, suicidal thoughts) due to their participation in chemsex. Respondents also experienced physical problems (e.g., sleep disorder, loss of consciousness, anal fissure, hallucination, etc.) (23,6%), sexual problems (21,3%), relationship problems (18,7%), and financial problems (8,4%).

Despite the above mentioned harms, no research has yet been done on the specific needs of these Belgian users. There was, however, a recent survey study (*n* = 52) among Belgian sex workers who engage in chemsex [34]. Results of this study show a need for access to reliable information, anonymous and free medical care, and psychological support to manage withdrawal.

A few other studies on information and care needs have been carried out in other European countries. A qualitative study (in-depth interviews) was conducted in South London, with the aim of understanding harm reduction needs among 30 GBMSM [35]. The study results showed two main needs: (1) the provision of reliable and non-judgmental information about safe drug practices, and (2) cooperation and exchange of knowledge between sexual health clinics and drug treatment facilities to close the gap of the existing support systems. Due to the combination of sexual and drug-related risks, sexual health clinics have few adequate answers to questions on drug use, while drug treatment centers generally lack expertise on sexual health problems and/or the gay subculture [2,36,37]. This need for the establishment of interdisciplinary and interinstitutional cooperation, and accessible information, was confirmed by a literature review from 2016 carried out by Pakianathan et al. [38]. Another finding from this literature review suggested the need for a non-judgmental approach by healthcare workers, especially because not all GBMSM disclosing their chemsex use have a problem that needs further support or referral. A qualitative study in Singapore [14] and an online survey in Great Manchester [39] also showed this need for a safe space and non-judgmental attitude. Especially because underlying factors such as shame, stigma, and the punitive nature of local drug laws act as a barrier to seeking professional help, or adhering to certain harm reduction strategies [14,15]. A previous qualitative study (in-depth interviews with 30 GBMSM) examining social norms relating to chemsex also suggested the existence of within-group stigmatization. More specifically, negative attitudes are often held regarding certain drug or sex practices, such as the use of crystal methamphetamine and injection drug use (“slamming”) [40]. This stigmatization of certain behaviors can also act as a barrier for these subgroups of GBMSM to seeking harm reduction information or accessing services [35]. Finally, a recent quantitative study in the Netherlands showed that almost one in four (23%) of GBMSM practicing chemsex stated a need for professional counselling, with a special interest in the following topics: increasing self-control, safer use of drugs, reducing sexual health related risks, and drug dependence [41].

As chemsex behavior (drugs and sexual consumption patterns), as well as the policies and possible support provided for this population varies greatly according to location [33], we wanted to gain more insight into the local chemsex context. This study aimed to understand current: (1) risk reduction practices before, during and after a chemsex event; and (2) information and care needs of GBMSM who engage in chemsex. The mapping of current risk reduction practices provides us with an important insight into the existing preventive and risk-taking behaviors of this group of GBMSM. This information will guide more appropriate support, and the possibility to better meet the needs of this key population in the future. We decided to carry out in-depth interviews to gain insight into the respondents thoughts and perspectives, and to provide more detailed information than what is available through other data collection methods, such as surveys or questionnaires [42]. Semi-structured interviewing also allows for more flexibility, such as adjusting the order of questions or allowing elaborating further on a topic mentioned by the respondent [43]. Finally, open-ended questions gives the respondent the opportunity to share their own experiences, which may reveal new behaviors. On the contrary, quantitative research only measures practices and needs that are known or expected.

To our understanding, this is the first qualitative study that focuses on harm reduction practices used by GBMSM who engage in chemsex. One previous German study focused on the use of crystal methamphetamine in sexual settings among GBMSM [44]. This study showed that most respondents use harm reduction practices. The most commonly used practices are: drinking enough non-alcoholic beverages, making sure to get enough sleep after consumption, and having enough lubricant available. Harm reduction practices seemed to be particularly employed among those who injected crystal methamphetamine, with measures such as: providing their own material and using clean needles. EMIS 2017 [45], a survey among GBMSM in Europe, also asked respondents about precaution behavior, but only related to sexual health. The following four practices were considered: taking antiretroviral drugs, sharing HIV information, using condoms, and being vaccinated. Further research using qualitative methods could help expand our current understanding of GBMSM preventive behaviors.

This study is part of a broader project, called “Chemified”, initiated at the Institute of Tropical Medicine (ITM) in Antwerp, Belgium. This project was set up to address the current lack of evidence-based support tools for chemsex-related issues by applying the promising field of mobile health interventions. The ultimate goal of this project is to develop a mobile phone application to support chemsex users in reducing the risks associated with chemsex, which is elaborated elsewhere [37]. This study describes the first step of the project, where we describe the experiences and needs of the local GBMSM community engaging in chemsex. This will ensure the mobile intervention contains relevant information, responds to existing needs, and sets achievable goals [46,47].

## 2. Materials and Methods

### 2.1. Study Design and Respondent Recruitment

A qualitative explorative study was conducted with semi-structured interviews among 20 self-identified GBMSM between the ages of 26 and 69 years old, between January and March 2020.

A mix of four recruitment strategies was used to reach our sample of 20 respondents. Initially, we chose to only recruit respondents through inviting members via the network of partnering organizations (*n* = 4) (expertise centers for sexual health and drugs). This strategy did not yield a sufficient number of respondents, which led to the implementation of other strategies: recruiting patients from a local HIV/STI/PrEP outpatient clinic (*n* = 12), announcing the project via posts on the partnering organizations Facebook pages (*n* = 3), and a snowball sampling approach at the end of each interview (*n* = 1).

Eligibility criteria included: being at least 18 years old; self-identifying as GBMSM; being able to express oneself in Dutch or English; and having intentionally used drugs (crystal methamphetamine, mephedrone, GHB/GBL, XTC/MDMA, cocaine, NPS, and/or ketamine) to have sex within the past 12 months.

### 2.2. Procedures

C.H. carried out a literature review on chemsex and mobile health interventions. This resulted in a matrix with chemsex related behavior and resulting risks, based on the intervention mapping approach [46]. The matrix contained “determinants”, “chemsex related behaviors”, “resulting risks and harms”, and potential “intervention components” to tackle these risks and harms. The matrix is added as Table S1 (Supplementary Materials). Based on this matrix, the project team formulated the questions related to harm reduction practices and mobile health intervention components (as part of the larger “Chemified” study, and with the aim of developing a mobile health intervention). The questions about information and care needs were formulated in close collaboration with the Flemish expertise centers for sexual health (SENSOA) and drugs (VAD). The final interview guide was then approved by the project team and the Ethics Committee.

The first three interview transcripts were read through by T.P. and C.H. in order to improve the interview guide. There were no new topics added, only follow-up questions were refined. This updated interview guide was approved by the project team. The interview guide can be found in Supplementary Materials (Questionnaire S1). This guide provided an overview of questions and themes that needed to be addressed during each interview. The questions and order of these questions was largely fixed, but there was the possibility, however, to deviate from the established structure by asking follow-up questions, depending on the respondents’ answers, or to change the order of the questions according to the natural flow of the conversation.

Interviews were conducted in Dutch or English either at ITM (*n* = 14) or online (*n* = 6). The reason for conducting online interviews was twofold. First, due to privacy reasons, two respondents only wanted to meet through a videocall during their working hours. The other four interviews were held online due to the COVID-19 lockdown, making it impossible to conduct in-person interviews. Respondents provided written informed consent prior to the interview and received €25 remuneration for participation (time and travel costs). On average the interviews took between 1 and 2 h.

The study was approved by the ITM’s Institutional Review Board (IRB) on 6 December 2019 (ref 1344/19).

### 2.3. Data Analysis

The interviews were conducted by the author C.H., who is a sociologist, and is trained in qualitative research methods. All interviews were digitally recorded and transcribed verbatim. All identifiers were removed from the transcripts to safeguard the respondents’ anonymity. After transcribing the interviews, NVivo 12 qualitative analysis software was used to support the management of the textual data, and to organize the codes assigned to the transcripts. A preliminary codebook with main categories was developed based on the preparatory literature review and interview guide, these included: risk reduction practices (before, during, and after a chemsex event), and information and care needs. During the analysis of the interview transcripts, these main categories were complimented by other themes, subthemes, and codes (inductive).

The interview transcripts were analyzed thematically according to the six phases described by Braun and Clarke [48]. First, all transcripts were thoroughly read in order to become familiar with the data. Comments or remarkable findings were also noted during this step. In phase 2, all transcripts were re-read to assign initial codes to the data. Following this, codes were grouped to form themes and subthemes (3). For example, the main category “harm reduction practices prior to a chemsex session” consists of the sub-theme “personal habits”, with different codes, such as “stay well hydrated”, “avoiding alcohol”, “taking vitamins”, and “eating sufficiently”. The themes formed during the previous phase were re-examined and sharpened (4). The entire dataset was re-read again to further refine the themes. Any additional data that was missed before was assigned to themes. During phase 5, the final themes were defined (5). The final phase consisted of producing this manuscript (6).

## 3. Results

The categories emerging from the final coding session reflected the structure used to discuss the research findings: harm reduction practices prior to the event, harm reduction practices during the event (drug-related practices, sex-related practices, mutual support), harm reduction practices after the event, and needs of chemsex users (reliable information and healthcare support).

Quotations were used to provide sufficient evidence to support the findings.

### 3.1. Respondent Characteristics

The respondents age ranged between 26 and 69 years (mean = 42.65; median = 43). Most of them lived in Antwerp (*n* = 18). The majority of respondents (*n* = 14) were employed. XTC/MDMA and GHB/GBL were the most commonly used types of drug, followed by crystal methamphetamine, mephedrone, NPS, cocaine, and ketamine. Intravenous injection of drugs (“slamming”) was reported by eight respondents. The number of years actively participating in chemsex ranged from 1 year to 28 years, with a mean of 8 years. The frequency of participation in chemsex differed from three times per week, to once every two months.

A detailed overview of these findings is provided in Table 1.

### 3.2. Harm Reduction Measures

In this section, the respondents’ harm reduction practices are presented. These results, thus, only presents findings from respondents who reported harm reduction measures, since there was also a minority of respondents (*n* = 4) taking few or no harm reduction measures. These respondents participate spontaneously and responsively in chemsex, and rarely consider possible precautions, consequences, or risks. When looking at Table 1, this group can mainly be found in the following categories: professional status “unemployed”, “injection drug use within the last 12 months”, frequency chemsex “more than once a week”, and the use of crystal methamphetamine.

**Figure 1:**
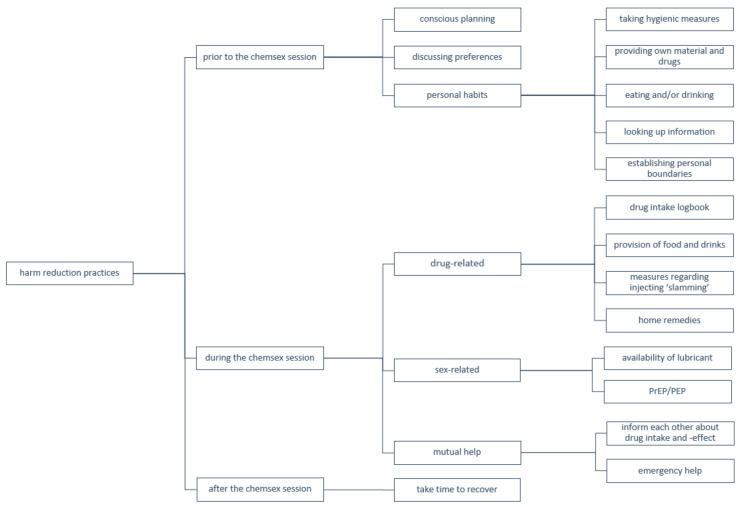
Schematic overview of harm reduction practices.

A schematic overview of harm reduction practices is provided in Figure 1.

#### 3.2.1. Prior to the Chemsex Session

The majority of respondents stated, when initially asked, that they did not take any preparatory measures before leaving to a chemsex event. As this respondent described:

“From the moment I see my mate, and he says something like “Come on, we’ll put a slam tina”, then it’s like: “Okay let’s go.” And suddenly you are so horny and you start looking on Grindr, and then you’re gone for God knows how long.”

(Respondent 2, 28 years old)

When the respondent could not list any preparatory measures, the interviewer asked more specific questions. A few examples of such questions: “Do you prepare yourself physically or mentally in the days or hours before a chemsex session?”, “Are there certain things you should not forget to bring to a chemsex session?”, “Do you have certain requirements to decide whether or not you go to a chemsex session?”, etc. Upon asking these questions, some measures were mentioned, yet preparation was rather limited. The cited practices will be discussed in the following paragraphs.

The majority of respondents (*n* = 15) tried to schedule chemsex sessions to avoid interference with their professional obligations. They worried that their work situation would adversely be affected by their participation in chemsex. For this reason, some men limited their chemsex engagement to the weekends in order to have sufficient time to recover physically and mentally. When they decided to meet up during the working week, they limited themselves (e.g., deciding in advance how long they would stay, not taking certain drugs).

“I usually meet up on a Thursday night. I don’t work on Fridays, so this means that I have Friday, Saturday and Sunday to recover. My work is very important to me, and I don’t want to look like a zombie the next working day.”

(Respondent 8, 45 years old)

Many men (*n* = 11) also described certain preferences regarding setting and attendees when scheduling a chemsex session. Most respondents preferred a small group of people (e.g., five people), because it is easier to monitor everyone, it is more intimate, it creates more trust between the attendees, and makes it easier to contact attendees afterwards for partner notification (e.g., in case of STI).

“In small groups, it gets less out of hand. When there are more people you notice that the party gets more chaotic and unmanageable”.

(Respondent 19, 48 years old)

Respondents (*n* = 11) also discussed certain issues with the attendees beforehand: which substances will be used (with particular attention to crystal methamphetamine), if intravenous injection will take place, what kind of sexual contact one is looking for (insertive, receptive, both, with or without a condom) and practical matters (possibility to spend the night, possibility to take a shower, if certain things need to be brought along (e.g., drugs, snacks, soft drinks, etc.).

“The first question is always: which chems do you have?”

(Respondent 7, 53 years old)

Other preparatory measures in the run-up to the event remain limited, but some men mentioned personal habits in order to mitigate the risk of complications due to chemsex. This included the following: taking hygienic measures (*n* = 14) (e.g., showering, shaving, anal douching), paying extra attention to what they eat and/or drink hours before the event (*n* = 13), bringing their own material and drugs (*n* = 12), looking up information regarding drug- or sex-related risks (*n* = 6), and establishing personal boundaries (*n* = 6).

“I’ve looked on the internet for certain things, I’ve done that. I don’t know which website it was, but a website with information about different drugs”.

(Respondent 13, 37 years old)

#### 3.2.2. During the Chemsex Session

##### Drug-Related Harm Reduction Practices

The vast majority of men (*n* = 18) mentioned that a logbook was kept with an overview of the drugs taken by each man attending the chemsex session. They especially highlighted its importance when taking GHB/GBL, as accurate dosing of this drug is critical. Usually this overview is written down on a piece of paper in a separate and easily accessible room, often the kitchen. In this logbook the name of the user, time, and (sometimes) dosage are noted. The logbook is useful for the user himself as well as for the others, as illustrated below by one respondent:

“When you suddenly say: “Shall we take another dose?” You take a look at the paper and see: “Oh it was only half an hour ago. No, not yet.” So that alone prevents so much. But also for others, when things go wrong you can just go and look: “What did he take?” That’s actually the most important thing.”

(Respondent 14, 26 years old)

Eight respondents identified the host of the chemsex event as the person responsible when something goes wrong (e.g., in case of drug overdose). For this reason, the host limits alcohol and drug consumption to be able to monitor everyone. He is therefore also the one who usually writes down the information in the logbook.

“When it is at our home, I always keep an eye on things. Usually the host is a bit more responsible, I guess. (…) That’s why when I go to a party somewhere else, I’m a bit more involved with the party itself.”

(Respondent 13, 37 years old)

In addition to filling in the logbook, the host provides food and drinks. In terms of food, this usually involves fruit, candy, and other small snacks. However, the feeling of hunger and thirst is usually so low due to the drugs, that no one or only a few people consume it.

Thirdly, respondents who inject drugs (*n* = 8) mentioned several measures to reduce associated risks: injecting with clean needles (*n* = 7), disinfecting (*n* = 6), being injected by someone who has more knowledge or experience (*n* = 4), and making sure the drug has dissolved completely (*n* = 3).

“With slamming it is important to bring your own needles. You can’t trust anyone in that regard.”

(Respondent 6, 27 years old)

Respondents (*n* = 8) also mentioned certain home remedies, which are supposed to help when someone feels unwell. For example: consuming active carbon, chocolate, tonic, fruit juice, or milk because they have a detoxifying effect.

Most men (*n* = 14) stressed the fact, however, that it is difficult to adhere to the above mentioned strategies when under the influence of drugs:

“Your standards fade when you take crystal meth. That’s what it does to you, blurring standards. So does GBL. It makes you push your limits, but that’s not always a good thing”.

(Respondent 2, 28 years old)

“I have an occasional fist date with someone, and he actually bleeds a lot, uhm, we even have to put towels underneath. But it doesn’t bother me and it doesn’t bother him, and that’s for sure the effect of the 3-MMC. Because I swear I wouldn’t be doing that if I hadn’t taken chems”.

(Respondent 9, 52 years old)

##### Sex-Related Harm Reduction Practices

Very few respondents (*n* = 3) mentioned sex-related harm reduction practices. When asked about harm reduction practices, respondents first talked about measures related to drug use. Only later, when explicitly asked, people thought about ways in which they tried to avoid sex-related risks.

Most men (*n* = 13) do not ask for information or communicate in any way about HIV status and/or last STI test, because the other is perceived to be unreliable. Respondents realize that those present have changing sex partners, so they are aware that there is a risk that someone has contracted an STI or HIV. Respondents therefore place this responsibility solely on themselves. This manifests mainly in taking PrEP, PEP, or antiretroviral drugs to prevent getting or spreading HIV. It is assumed that others also take PrEP when HIV negative, and antiretroviral therapy (ART) when HIV positive. However, the risk of contracting STIs is downplayed due to the availability of treatment to fully recover (in contradiction to HIV). In this context, respondents cited they do not use condoms when participating in chemsex.

“For STIs it’s very limited. Because first of all, you’re messing around with the condom. Also on the one hand a bit cornered by PrEP. We can’t win the jackpot anymore so, let’s go! We’ll see other STIs rise, sky high, but so be it. Those STIs are often considered as something you can get rid of easily. And, one pill or two more doesn’t matter either”.

(Respondent 5, 29 years old)

Finally, all respondents cite that lubricant is present, usually provided by the host. This was considered important because of the often long sessions. The lubricant was shared though, which in turn entailed other risks.

##### Mutual Help

Many respondents (*n* = 17) also described the element of care. Not only self-care as described above but also caring for each other. This was described in different ways. Some men (*n* = 9) cited that they would inform others about correct dosage, combinations, and effect of certain drugs. Furthermore, respondents (*n* = 10) highlighted how they kept an eye on each other, and helped others when things went wrong.

“What’s a pretty common thing when someone isn’t doing well, is putting him in the shower. Try to wake him up. Not too cold, not too hot water. If that doesn’t help, I always keep checking his breathing”.

(Respondent 8, 45 years old)

Conversely, respondents (*n* = 18) also admitted that they didn’t fully trust others to take care of them. The expression “You are responsible for yourself” and “You cannot rely on anyone” were often cited by respondents:

“I always hope, if I get a little too high myself—that they’ll take care of me too. But sadly, I’m not so sure about that”.

(Respondent 15, 59 years old)

One reason for this lack of trust is the great reluctance that respondents (*n* = 15) experience to notifying emergency services during a chemsex event. This is due to the fear of police showing up and possible persecution. This hesitation to notify emergency services when help is needed, creates feelings of distrust of the other.

“Um, the fact remains, drugs are illegal. So yeah, if it really goes wrong, calling an emergency number… that’s a very, very delicate matter”.

(Respondent 14, 26 years old)

Furthermore, respondents (*n* = 9) expressed concerns about the lack of knowledge among co-users. The overall knowledge about substances used, and their associated risks, is perceived as low. This can lead to the dissemination of misinformation when trying to inform others.

“There are a lot of people who think they know everything, but lack the right information. They claim they know it all and would even get angry if people question it”.

(Respondent 3, 26 years old)

Others (*n* = 7) also pointed to peer pressure in this context. This can cause people to take more risks, such as taking more, or different, drugs than they initially intended. It also acts as a threshold for asking others for information, as one respondent described:

“I have to say, it’s a bit of a shame indeed. When you get into a setting like that, there’s a lot of showing off. Even though you don’t know anything, you’re not going to ask: “What’s that?” Cause then it’s like: “What are you doing here?” It’s a bit of a shame that you can’t communicate with someone like: “What chem is that? And what kind of effect does it have?””

(Respondent 20, 52 years old)

#### 3.2.3. After the Chemsex Session

Respondents (*n* = 14) reported taking time to recover physically and mentally from the chemsex session. During this period, which was often scheduled (as discussed above), three main strategies were used to deal with the negative after-effects: resting and catching up on sleep, doing mindless activities, and eating and drinking sufficiently.

“I try to sleep as much as I can. I tend to lock myself up at home at that moment. This already happened so frequently that I am beginning to know the course of things. The first day is like this, the second day like this and by the fourth day—it will take four days for me, then I’ll feel okay again”.

(Respondent 4, 58 years old)

On the contrary, there is a small group of respondents (*n* = 4) who did not take the time to recover, but instead took additional drugs or medication to soften the aftereffects and deal with the comedown.

“Yes, you can’t do anything and can’t get anything done. You get so tired of it that you take something again. And then the whole thing starts over.”

(Respondent 2, 28 years old)

### 3.3. Needs of Chemsex Users

**Figure 2:**
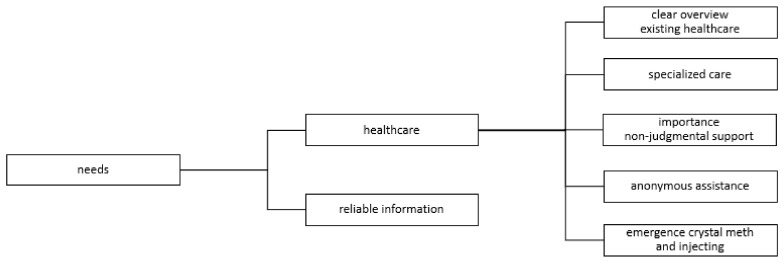
Schematic overview of information and care needs.

A schematic overview of information and care needs is provided in Figure 2.

#### 3.3.1. Reliable Information

When asked about chemsex users’ needs, the large majority of respondents (*n* = 16) stressed the importance of reliable information. When searching for chemsex-related information one often finds contradictory results, as one respondent mentioned:

“All you do now, if there’s something you want to know, is to Google it. But yeah (…) One says A and the other says B so, which one is true?”

(Respondent 9, 52 years old)

Therefore, there is a need for a gathering place of reliable and easily accessible information. In this way it could also be consulted on the spot during a chemsex party.

The lack of information regarding emergency help was especially pointed out (*n* = 15). Most respondents admitted they do not know how to assist someone in case of an overdose (or what not to do):

“That when things go wrong, I think people know they should call 112, but in other light situations, there’s a lot of uncertainty and ignorance.”

(Respondent 3, 26 years old)

In addition, more than half of the respondents (*n* = 14) highlighted the need for accessible information about harmful drug combinations. There seems to be a great deal of uncertainty about the effects of combining different substances, in particular with regard to new psychoactive substances.

“Above all, mixing is also an important issue. I mean, can I mix crystal meth with ketamine? But can I also use crystal meth and 3-MMC together? Can I take an XTC pill with it? Maybe two glasses of GBL, is that all right? And what about poppers? How does that relate to each other?”

(Respondent 18, 28 years old)

Other useful information respondents mentioned concerned: drug effects, correct dosage of drugs, information specific to injection of drugs, chemsex related risks, safer sex guidelines, and symptoms of STIs.

#### 3.3.2. Healthcare Support

Most respondents (*n* = 14) expressed a need for a clear overview of existing (drug and sexual) healthcare (and peer support) structures. Many do not know where to turn for their chemsex-related questions and concerns. As this respondent stated:

“I don’t actually know because I’m here because I coincidentally told [name doctor] at the right time—I didn’t even know about the existence of certain services”.

(Respondent 10, 39 years old)

Similarly, respondents (*n* = 7) stated that it was difficult to find professionals to talk to for both drug-related and sex-related questions. Specialized counselling would therefore provide an appropriate answer.

“Every psychologist I ended up with had more experience in one field than another. It turned out to be very hard to find one specialized in addiction combined with sex. Uhm, yeah, I found zero good counselling”.

(Respondent 4, 58 years old)

Furthermore, respondents (*n* = 9) emphasized the importance of non-judgmental support. According to the respondents, there is still a lot of shame and stigma surrounding chemsex because it entails the use of drugs and certain sexual practices. To open up about participation in chemsex, it is necessary to create a safe space where people can talk openly about their use without any judgement. A respondent shared his experience:

“You can only talk about it when you’re in a safe environment. At one point I had a doctor for my usual check-up and I felt that he absolutely did not agree with it. He didn’t literally say that, but I felt that he thought it was my own fault”.

(Respondent 18, 28 years old)

Accordingly, more than half of the respondents (*n* = 11) also highlighted the need to preserve their anonymity. As mentioned above, many men have experienced shame, or fear being judged or stigmatized by healthcare professionals. In addition, chemsex was mostly hidden from family and friends. In this regard, respondents described ways in which they safeguarded their real identity:

“You never use your real name. Uhm, I also never use my personal cell phone number, but always that from work. My phone is also turned off at home. So yeah, everyone uses their own methods.”

(Respondent 17, 49 years old)

Lastly, there is a concern about the emergence of crystal methamphetamine and injecting (“slamming”). More than half of the respondents (*n* = 13) expressed concern about the role crystal methamphetamine is playing in chemsex sessions. They feel that the drug has become increasingly widespread, and that its use has become normalized in recent years. Injecting drugs (not only crystal methamphetamine) is also becoming more and more accepted and widespread.

“It’s getting popular really fast. So I would say; keep that in mind. Because there is no help for it. I’ve noticed it myself with my crystal meth comedown. There is no counter medication for it so you have to try to get rid of it with pure willpower. I haven’t succeeded yet.”

(Respondent 6, 27 years old)

## 4. Discussion

We want to stress that the results obtained from this qualitative study are not intended to explain the experiences and needs of all GBMSM participating in chemsex. Interviewing 20 GBMSM allowed us to explore a wide range of harm reduction practices and needs, but they are probably not representative of, nor generalizable to, all chemsex users.

We identified several trends in harm reduction practices. Most chemsex users (*n* = 16) implement a number of strategies to manage the risks associated with chemsex. They tend to consciously plan chemsex sessions according to their personal schedule, gather information about safer sex and drug use, communicate drug- and sex-related preferences in advance, take time to prepare themselves in the hours leading up to the event, and set clear personal boundaries. During the chemsex session the focus lies mainly on reducing drug-related risks. The most commonly used measure is keeping a logbook in which the drug intake of each person is recorded. After participating in the chemsex session one takes enough time to recover physically and mentally. For this group of respondents harm reduction practices seem to be a well-established part of their participation in chemsex.

Remarkably, there was also a group of respondents (*n* = 4) who mentioned hardly any, or even no, preventive behaviors. Their decision to attend a chemsex event was more spontaneous, ad hoc, and opportunistic. This, of course, leaves little time to take preparatory measures. However, consistent with their lack of preparation, they only take minimal precautions (limited to drugs) during the event, and indicated that they take drugs again to reduce their comedown after a chemsex event.

Based on these findings, we can differentiate two groups of users: the planned user and the impulsive user. Most respondents use a variety of strategies to minimize chemsex associated risks, and only a minority took little or no preventive measures. Our results share similarities with Bourne et al.’s [35] findings from 30 in-depth interviews, stating that respondents could be divided in three categories of exposure and recognition of harm: GBMSM who feel in control of their chemsex use, and employ harm reduction practices to manage their frequency, dosing, and dependency; respondents who felt their drug use was problematic and noticed negative effects; and a third category of respondents who had a problematic relationship with chemsex, but did not recognized it as such themselves. These different user profiles support previous findings that engaging in chemsex does not necessarily lead to the development of extensive harms for every user [49,50]. Some users feel in control of their use, are generally satisfied with their participations, and experience (relatively) few negative effects, i.e., “planned users” [15,41,51].

Another group of chemsex users do not take these preventive measures. Their unplanned (“impulsive”) decision process to participate in chemsex events (with an accompanying lack of preventive measures) may expose them to increased health risks. When looking at the interview data, “impulsive users” are more likely to: be unemployed (*n* = 3), slam chems (*n* = 3), use crystal methamphetamine (*n* = 4), and participate in chemsex more than once a week (*n* = 3).

These findings seem to be contradictory with the recent study by Schecke et al. (2019), which focused on GBMSM who use crystal methamphetamine in a sexual context [44]. This study showed that a group of crystal methamphetamine users, especially those who inject (potentially causing the most severe health impact) implement the highest level of preventive measures. This may imply that not the use of crystal methamphetamine as such, but an underlying (psychological or personality) factor may lead to lose one self in chemsex with a severe health impact. From this point of view, the use of crystal methamphetamine among this latter group is a consequence, rather than a cause, of “impulsive” chemsex use.

Despite the wide range of mentioned harm reduction practices, certain factors complicate the adherence to intended measures: the effects of drugs, peer pressure, and feelings of distrust. When under the influence of drugs, individuals suddenly no longer behave in line with their predetermined boundaries. They report losing themselves completely in the intense feelings of sexual arousal and disinhibition. Peer pressure prevents GBMSM from asking for information during a chemsex party, and pushes them to go beyond predefined limits. Feelings of distrust sharply contrast with taking care of each other, often mentioned by chemsex users. It seems that chemsex users rely mainly on themselves. This was mentioned during conversations about HIV/STI (“you are responsible for taking PrEP”), by providing one’s own material and drugs during chemsex sessions, (not) calling the emergency services in crisis situations, and the spreading of misinformation.

This project also aimed at identifying the information and care needs of Belgian chemsex users. Our results suggest that GBMSM who engage in chemsex have a hard time finding reliable and easily accessible information. This is consistent with the findings from a recent Belgian survey among sex workers who engage in chemsex (34), and a qualitative study from 2015 on the harm reduction needs among GBMSM chemsex users in South London [35]. Next to information needs, there are still clear gaps regarding knowledge about care and (peer) support. Respondents do not know where to turn to for chemsex-related questions or help. When GBMSM engaging in chemsex look for help, they usually do not find appropriate counselling for their problems, and are often repeatedly referred from one organization to another. There is thus a need for an overview of existing healthcare services, and more specialized help focused on chemsex issues. Other studies also showed this demand for specialist chemsex services and more efficient referral between sexual health clinics and drug treatment facilities [2,36,37,38]. These findings are in accordance with findings from a quantitative study (*n* = 150) aimed at exploring the demand for, and availability of, chemsex services in the UK [52]. This online survey was distributed among sexual health clinic healthcare workers, and reported a need for chemsex training (99%) and a local chemsex specific service (81%). Furthermore, chemsex users too often experience a value judgment from a healthcare professional. It is therefore necessary to guarantee a safe environment in which assistance is provided in a non-judgmental manner, and where it is possible to maintain anonymity. These needs for a safe and value-neutral space are in line with previous studies in other countries [14,15,39].

Chemsex is often experienced as a hidden life where anonymity and secrecy are important. This is caused, and reinforced, by feelings of shame, stigma, and existing legal barriers [5]. This is also confirmed in the analysis performed for this project. Respondents thus belong to a hard-to-reach key population, as it is difficult to talk openly about drug use and participation in chemsex. These factors imply difficulties in respondent recruitment. Nevertheless, we succeeded in recruiting sufficient GBMSM engaging in chemsex to get a snapshot of their strategies to mitigate risks, and of current needs for information and care.

Some limitations are worth noting. First, respondents were asked to share experiences about drug use and sexual behavior. Self-reporting about these potentially stigmatized behaviors could have led to recall bias and social desirability bias. It is thus possible that these biases may have had an effect on the collected data. Second, despite an intensive search through various channels, we were unable to recruit young GBMSM who participate in chemsex. The youngest respondent was 26 years old. Another limitation of the study was the choice of both face-to-face and online interviews. We offered the option for online interviews due to the privacy concerns of respondents and the COVID-19 measures, but we acknowledge this might have had an impact on the study results, as interviewing through Skype has its own limitations (e.g., it is impossible to observe all of the respondent’s body language, possible network problems affecting interview continuity) [53]. Finally, as already indicated, the study results should not be generalized to the whole GBMSM community who participate in chemsex, due to the size of our sample.

## 5. Conclusions

Our findings suggest that most GBMSM who participate in chemsex take harm reduction measures to mitigate related risks. Harm reduction practices seem to be a well-established part of their participation in chemsex. The most practiced measures constitute of: consciously scheduling the chemsex session, discussing preferences, having personal habits to prepare oneself, keeping a drug logbook during the chemsex session, the role of the host as supervisor, trying to assist each other, and taking time to recover. Adherence to these practices is often interfered with by the effects of drugs, peer pressure, and feelings of distrust towards others present at chemsex sessions.

Future studies should elaborate on which factors make a person belong to the group of “impulsive users”, as they do not take any, or very little, preventive measures. They are therefore the most exposed to risks.

Recommendations for current chemsex support and care can be made based on the mentioned needs. Chemsex users specifically attached a great deal of importance to making the issue discussable, both with health professionals and people around them. However, they experience fear of being judged for their participation in chemsex. It is necessary to create a safe environment which is value-neutral, where people can talk about their use. Accordingly, options must also be created for anonymous assistance. There should also be more focus on specialized assistance. It is necessary to make healthcare professionals aware of the issue, and to train them on how to best support or refer people with these questions. In addition, attention should be paid to the emergence of crystal methamphetamine and slamming. Finally, there is a need for a gathering place of reliable information that is easily accessible at the same time.

The applied risk reduction practices with associated thresholds (i.e., effects of drugs, peer pressure, distrust) and information and care needs provide the starting point for the creation of a mobile health intervention to support this group of GBMSM. The mobile health intervention should meet some of the mentioned needs and risks.

## Figures and Tables

**Table 1:** Characteristics of gay, bisexual, and other men who have sex with men (GBMSM) who participated in the in-depth interviews (*n* = 20).

**Age**	***n***
<25 years	0
25–29 years	6
30–39 years	3
40–49 years	4
50–59 years	6
≥60 years	1
**Professional Status**	***n***
Employed	14
Unemployed	4
Student	4
Retired	4
**Drugs Used in Previous 12 Months**	***n***
XTC/MDMA	15
GHB/GBL	13
Crystal methamphetamine	11
Mephedrone	9
New psychoactive substances (NPS)	8
Cocaine	7
Ketamine	5
**Injection Drug Use**	***n***
Never	12
Within the last 12 months	7
>12 months ago	1
**Years Active Chemsex**	***n***
1–2 years	3
3–5 years	8
6–10 years	5
11–20 years	3
≥20 years	1
**Frequency Chemsex**	***n***
Daily	0
More than once a week	3
Weekly	9
Monthly	5
>monthly	3

## Supplementary Materials

The following are available online at https://www.mdpi.com/1660-4601/17/23/9081/s1, Table S1. Matrix, Questionnaire S1: Interview guide.
