# Supplementary material for: Harm Reduction Practices and Needs in a Belgian Chemsex Context: Findings from a Qualitative Study
Source: Int J Environ Res Public Health. 2020 Dec 4;17(23):9081. doi: 10.3390/ijerph17239081 (PMC7730975; doi:10.3390/ijerph17239081)
Supplement: Supplementary file 1 [file ijerph-17-09081-s001.pdf]

Table S1. Matrix**.**

DETERMINANT	BEHAVIOUR	RISK	INTERVENTION
Little knowledge about drug related risks	Polydrug use	Dangerous interactions of different drugs;	- *Information and advice* about substance use (general harm reduction advice): e.g. combo chart, cfr. Materials Safe ’n Sound and Never Waste a Great Party campaign
	Injecting drug use	Higher risk to addiction and infectious diseases	- How to use and clean your paraphernalia
	Using high doses, extensively redosing	Higher risk for overdose, exhaustion, sexual risky behavior	- Informing and raising awareness
	Sharing user equipment (needles, straws, …)	Higher risk for infectious diseases.	
	Having sex with partner that is unconscious	Not respecting consent, risk for accusation of rape, …	
	Not sleeping a lot	Sleep deprivation, acute irritability, depression, memory loss (forgetting Prep medication), psychosis, paranoia, anxiety, etc.	- How to *prepare* for such a weekend?; how to deal with the coming down period afterwards?<br>- *Monitoring mental health*; diary? How are you feeling today?

	Not eating enough (food deprivation)	Exhaustion, losing weight, …	- Push notifications *with reminders to take medication*
	Extensive, long sexual sessions with multiple people (driven by stimulant use)	Increased risk of STDs; Psychosocial harms: sexual selfishness, prioritizing chemsex over social engagements, work or other obligations.	- *Information*: signals relating to possible problematic drug use.<br>- General intervention: information guide on how to organise a chemsex party
Loneliness	Search for connection through dating apps	Search for connection turns into chemsex connection: Dating apps make drugs and sex easily accessible	- Virtual *peer support group*<br>- (temporarily) *blocking apps*
Stigma/trauma/internalized homophobia/shame	Use of drugs (as a means of coping with sexual identity issues)	Drug use as a coping strategy, which could lead to drug dependency/addiction	- *Chatbots, gateway to assistance* (hulpverlening) → provide a safe space where men can talk about the use of chems without being stigmatized or judged<br>- *Self-assessment*<br>- Provide a *holistic treatment*: looking at sexual identity, drug use and psychological and psychosocial issues combined
Dissatisfaction with sex life and low self-esteem	Drug use in a sexual setting (using drugs for sexual enhancement)	Sexual risk-taking behavior (fisting/condomless sex/increased number of sexual partners/long sexual sessions)	- Suggestions for healthier alternatives (nudging)
		Increased risk at contracting STI or HIV	- Push notifications with reminders to get tested

HIV status	HIV positive men are more likely to engage in high risk chemsex related behaviors	Increased risk of contracting/spreading STI/HIV<br><br>Forgetting to take HIV medication	- Creating a *risk profile*: providing more targeted help. State somewhere whether you are HIV positive or not (risicobevraging bij het aanmaken van je profiel op de app?)

Questionnaire S1: Interview guide

PHASE 1: harm reduction practices & needs

Background

- When you think back on the first time you engaged in chemsex, what motive(s) did you act on? What kind of feelings/ideas/thoughts occurred to you?
- Since when do you participate in chemsex?
- How often do you participate in chemsex?
- When was the last time you participated in chemsex?
- Which chems do you use?

Harm reduction practices

- Can you describe a typical week in which you engage in chemsex?
- How do you prepare for a chemsex event, the days or hours before?
- What preventive measures do you take during a chemsex event to prevent HIV, hepatitis C and other STIs?
- What preventive measures do you take during a chemsex event to prevent drug related harms (e.g. drug overdose, drug combinations)?
- How do you experience the days after a chemsex event? What do you do to cope with or counteract the negative effects?

Information and care needs

- Have you ever looked for information regarding chemsex?
- Do you know where to find information about chemsex or chemsex-related risks?
- Did you find the information you were looking for?
- Do you know where to find chemsex assistance?
- Have you ever considered seeking help or support? Did you feel that you could use support or assistance at certain times?
- At which moments did you wish to receive certain support?
- How would you wish to receive this support?
- What was your experience with the support you received?
- What do you think is missing from the current (drug and sexual) healthcare offer?

PHASE 2: reviewing intervention components

- Suppose there is an app that would help you participate more safely in chemsex, what do you think it should consist of?
- What would prevent you from using the app? (thresholds)

Discussing intervention components that have not yet been mentioned by the respondent.

THEME 1: INFORMATION AND ADVICE

- Offering information and advice about drug use
    - Interaction between different drugs/medication
    - Safe drug use
    - What do you do in case someone overdoses at a chemsex event?

    - In case of an overdose, which information do you provide to the emergency services?
- Tips for a safer chemsex party/weekend,
  e.g..: How do you prepare yourself (eating enough, getting enough sleep, etc.)? How to deal with the ‘dip’ afterwards?
- Information about signals that indicate that recreational drug use is evolving into problematic drug use.
- A guide that helps people organize a safer chemsex party.
  e.g.: a template to properly monitor GHB intake of the attendees

THEME 2: SOCIAL NETWORK AND ASSISTANCE

- Chatbots (who can refer you to appropriate assistance if needed)
  e.g.: show examples of chat bots that currently exist in the field of online/mobile health interventions
- Virtual peer support group
  e.g.: a group of people who you can contact at any time, who understand what you are going through and can support you. Also show existing examples for support.
- Information and access to assistance
  e.g.: existing care systems in Flanders/Brussels, where do you need to go for which questions/problems/needs?

THEME 3: ATTITUDE AND SKILLS

- Monitoring your mental health/state of mind
  e.g.: via daily questions that are easy to answer: ‘How are you feeling today?’
- Goal setting and reflection
  e.g.: the possibility to indicate what your plans are during a certain period of time, the app subsequently checks what you have actually done. Short and longer term behavioral goal setting.
- Self-assessment
  e.g.: assessment that people may have more insight in own functioning and potential problematic chemsex use.

THEME 4: ADDITIONAL FUNCTIONALITIES

- Push notifications
  e.g.: with reminders to take certain medication, with reminders to get tested
- Location
  e.g.: indicating certain areas that may be risky for you, for example, the app can send you a message when you come near these areas
- Nudging: suggestions for healthier alternatives

THEME 5: CUSTOMIZED INFORMATION AND ANONYMITY

- What information are you willing to provide in order to create a profile?
  e.g.: your name, e-mail address, city where you live, location, etc.
- Creating a risk profile that allows to offer more targeted assistance. Important: the more information provided, the more personalized care can be offered.
- To what extent are you willing to provide certain information in exchange for more personal information and advice from the tool? (gives us the possibility to offer dynamic info instead of static). e.g.: filling out a questionnaire when creating a profile. Which periods you engage in chemsex (e.g. days in the week), which drugs you mainly use, what your triggers are (e.g. boredom, going out, being alone, when you experience stress), etc.
